# Massage protects skeletal muscle from injury during long-term heavy-duty exercise via integrin β1 and laminin 2 channels of basement membrane

**DOI:** 10.1186/s12906-023-04094-6

**Published:** 2023-07-26

**Authors:** Qingsong Liu, Songlin Jin, Lunyu Li, Liubu Ayi, Haili Ding

**Affiliations:** 1 Sichuan Academy of Medical Sciences & Sichuan Provincial People’s Hospital, Chengdu, China; 2 School of Sports Medicine and Health, Chengdu Sport University, Chengdu, China; 3 Insititute of Sports Medicine and Health, Chengdu Sport University, Chengdu, China

**Keywords:** Massage, α7β1 integrin, Laminin 2, Long-term heavy exercise, Overuse injury, Skeletal muscle

## Abstract

**Background:**

Massage is widely used in exercise-induced skeletal muscle damage (EIMD). It has been proven that massage can improve the morphology and function of damaged skeletal muscle in multiple ways. However, whether massage can protect skeletal muscles from injury during long-term heavy-duty exercise has not yet been determined.

**Methods:**

In this study, a rat model of overuse injury was established by eccentric running for 4 weeks, and pressing at constant pressure and frequency and massage were used as intervention methods to explore whether massage could protect skeletal muscle from injury through upregulating integrin and the basement membrane laminin.

**Results:**

The results showed that compared with the model group, the ultrastructure of skeletal muscle in the massage group was relatively complete and clear, and the maximum isotonic and tetanic contraction forces were significantly increased (*P* < 0.01). In addition, in the massage group, β1 integrin expression was significantly increased, p-FAK protein expression was decreased, and the co-localization of β1 integrin and the basement membrane laminin 2 was significantly increased (*P* < 0.01).

**Conclusion:**

Our study shows that during long-term heavy-duty exercise, massage can enhance the cell adhesion function mediated by integrin β1 and laminin 2 to protect skeletal muscle from injury and prevent the occurrence of overuse injury.

**Supplementary Information:**

The online version contains supplementary material available at 10.1186/s12906-023-04094-6.

## Background

To increase muscle strength and endurance in competitive sports, repeated heavy-load (eccentric) exercise training is often carried out. However, heavy-duty exercise (especially unaccustomed eccentric exercise) may cause exercise-induced skeletal muscle damage (EIMD), which mainly manifests as decreased muscle function and delayed-onset muscle soreness (DOMS). EIMD can generally be restored well by resting, but in competitive sports, outdoor sports, and physical labor, due to long-term repeated heavy-duty exercise, the injured skeletal muscles cannot rest adequately, resulting in overuse injury [1]. Therefore, there is a need to investigate how to protect skeletal muscle from injury during long-term heavy-duty exercise. Skeletal muscle injury caused by long-term repetitive training is considered a type of Bi syndrome in Chinese medicine, and its main pathogenesis is the closure and blockage of the meridians. Massage therapy in Chinese medicine can balance yin and yang, and unblock the meridians, which can relieve local muscle spasms, restore muscle function, and alleviate symptoms. Massage is widely used to promote the recovery of skeletal muscle fatigue and injury after sports training in competitive athletes. Massage can reduce the inflammation of damaged skeletal muscle and promote mitochondrial repair [2], improve DOMS [3], reduce muscle strength loss [4], and promote EIMD repair [4]. However, it is still unclear whether massage can protect skeletal muscles from injury during long-term heavy-duty exercise. The extracellular matrix (ECM) is a complex network of multiple proteins that encases skeletal muscle fibers, and is often described as a mechanical force-sensitive device. Integrins mediate cell adhesion function and sense the transduction of mechanical forces by linking actin and ECM, thereby avoiding skeletal muscle damage under stress. Previous studies have found that α7β1-integrin binds to the basement membrane laminin 2 to regulate mechanotransduction and prevent skeletal muscle injury [5]. In adult muscle fibers, β1D integrin is the predominant form and mediates stronger interactions with laminin [6, 7]. In muscular dystrophy mdx mice and in vitro experiments, increasing β1D chains simultaneously enhanced α7 integrin and α2 laminin amounts and protected skeletal muscle sarcolemma from damage [8]. In the present study, we explored whether massage can protect skeletal muscle from injury during long-term heavy-duty exercise by enhancing the functional pathway of cell adhesion mediated by β1 integrin and laminin 2.

## Materials and methods

### Experimental animals

Thirty-two 8-week-old SPF-grade healthy male Sprague-Dawley rats were purchased from Chengdu Dashuo Laboratory Animal Co., Ltd. (Qualification No.: SYXK (Chuan) 2020-030).

### Main reagents

Anti-integrin α7 was purchased from Santa Cruz Biotechnology (USA). Anti-integrin β1 was bought from GeneTex (Germany). Anti-FAK was obtained from Jiangsu Jinke Biological Research Center Co., Ltd. (China). Anti-p-FAK was purchased from Jiangsu Qinke Biological Research Center Co., Ltd. (China). Anti-β-actin was bought from Wuhan Aibo Biotechnology Co., Ltd. (China). Anti-Na^+^/K^+^-ATPase was obtained from Abcam (UK).

### Main instruments

The following instruments were used: a Finger-8-TR FSR film pressure sensor (Suzhou Changxian Optoelectronics Technology Co., Ltd., China), an HV1403 isothermal tissue and organ perfusion system (Chengdu Taimeng Software Co., Ltd., China), a QuantStudio TM3 real-time fluorescence quantitative PCR (RT-qPCR) instrument (USA), a 5200 chemiluminescence gel imager (Shanghai Tianneng Technology Co., Ltd., China), and an MK3 full-function microplate reader (USA).

### Grouping and exercise program

Thirty-two SPF 8-week-old male Sprague-Dawley rats were randomly divided into the following four groups: a blank control group (C), a long-term heavy-duty exercise group (M), a long-term heavy-duty exercise + sham massage group (G), and a long-term heavy-duty exercise + true massage group (T) (*n* = 8 rats per group). Rats were kept at room temperature with free access to water and food. All experimental protocols complied with animal ethics standards and were approved by the Ethics Committee of the Chengdu Institute of Physical Education (approval number: 2021-33). Long-term heavy-load treadmill exercise training was carried out by rats in the M, G, and T groups. Adaptive exercise was started in the first week. On days 1 and 2, the slope of the treadmill was 0°, the speed was 16 m/min, and the daily exercise time was 10 min; on days 3 and 4, the slope was − 5°, the speed was 16 m/min, and the exercise time was 15 min; on day 5, the slope was − 10°, the speed was 16 m/min, and the exercise time was 30 min. This adaptive exercise was followed by 4 weeks of heavy-duty exercise (6 days a week, rest on Sunday). In week 1, the slope was − 16°, the speed was 16 m/min, and the exercise time was 60 min; in week 2, the slope was − 16°, the speed was 20 m/min, and the exercise time was 60 min; in weeks 3 and 4, the slope was − 16°, the speed was 20 m/min, and the exercise time was 90 min.

### Massage intervention program

**Fig. 1:**
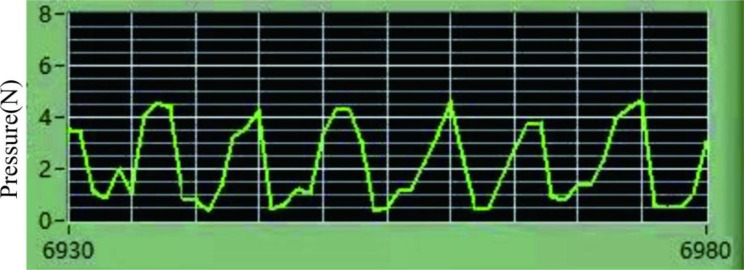
Quantification and monitoring of massage parameters

In the stage of formal heavy-duty exercise, rats in group T were given a massage 6 h after each exercise. Senior physicians specializing in massage in the Department of Traditional Chinese Medicine performed massage on the gastrocnemius muscles of both calves of the rats, once a day, for 5 min per massage on each side. Through wireless physiological recording combined with an FSR film pressure sensor, the massage parameters were maintained as follows: a pressing force of about 4 N and a frequency of 120 times/min. The parameter record is shown in Fig. 1. Group G was given a false push intervention through the use of a brush after six h of exercise. More specifically, as in the T group, the rats were fixed, and the massage physician then used a thumb-sized brush to stroke the hair above the rats’ triceps muscles at a frequency of 120 times/min without touching the skin. This was performed once a day for 5 min on each side. The rats in groups C and M were not given any interventions.

### Collection and storage of experimental samples

At 24 h after the last exercise, rats were anesthetized by an intraperitoneal injection of 1% sodium pentobarbital (30 mg/kg). The medial head of the left gastrocnemius muscle was isolated for in vitro muscle contraction force testing. The right gastrocnemius muscle was divided into three parts: (i) tissue fixed with 3% glutaraldehyde for transmission electron microscopy, (ii) tissue fixed with environmentally friendly GD muscle fixative for immunofluorescence staining, and (iii) tissue used for western blot and RT-qPCR analysis. Tissues were stored at − 80 °C.

### Test of isotonic force and tetanic force of isolated muscle

After the medial head of the gastrocnemius muscle was separated, it was quickly put into Krebs–Henseleit solution, and the tendons at both ends were bound with No. 0 medical surgical thread and fixed on the tension sensor. The initial contractile force was then used to electrically stimulate the tissue with a platinum wire and record the maximal isotonic and maximal tetanic force [9]. (i) The isotonic contraction stimulation conditions were as follows: single stimulation, voltage 10 V, wavelength 2 ms, stimulation interval 2 min, stimulate three times, record the maximum contraction force, and take the average value as the maximum isotonic contraction force value. After the single-contraction stimulation was over, the muscle tissue was allowed to equilibrate in a perfusion bath for 15 min before the tetanic force was measured. (ii) The tetanic stimulation conditions were as follows: continuous stimulation, voltage 15 V (isotonic stimulation voltage × 1.5), frequency 60 Hz, wavelength 2 ms, stimulation duration 60 ms, interval 2 min. The maximum contractile force was recorded three times, and the average value was taken as the maximum tetanic force value.

BL420 Data Acquisition and Analysis System (Chengdu TME Technology, Chengdu, China) was used to collect and analyze experimental data. As the calibration standard, the following value was used: (maximum contraction force − initial contraction force) / muscle strip cross-sectional area. Muscle strip cross-sectional area was calculated as follows: cross-sectional area (cm^2^) = wet weight / (muscle density × muscle length). The muscle density was 1.06 g/cm^3^ [10].

### Ultrastructural observation of gastrocnemius muscle observed by transmission electron microscope

After the fresh gastrocnemius muscle tissue was isolated, it was pre-fixed with 3% glutaraldehyde, re-fixed with 1% osmium tetroxide, dehydrated in acetone, infiltrated, embedded, and prepared into 50-nm sections. After staining with uranyl acetate and lead citrate, the samples were observed and photographed using a JEM-1400PLUS transmission electron microscope after magnification of 15,000 times.

### Western blot detection of FAK, p-FAK, integrin α7, integrin β1

The expression of target protein in tissues was determined by Western blot. First, 200 µl of RIPA lysis buffer was added to 20 mg of gastrocnemius muscle tissue, and the homogenate was smashed and lysed on ice. After centrifugation, the supernatant was taken, the protein concentration was determined, and equal amounts of protein were separated by SDS-PAGE. Next, proteins were transferred to PVDF membranes, which were blocked, incubated with primary antibody, washed, and incubated with secondary antibody. Membranes were washed three times with TBST, and protein bands were visualized with ECL luminescent solution with an exposure time of 1 min. Finally, the gray values of the bands were determined and analyzed using VisionWorks LS software.

### Gene expression of integrins α7B, α7X2, and β1D

Total mRNA of the gastrocnemius muscle was extracted using a kit, cDNA was synthesized, and then target fragments were amplified by PCR using the cDNA as a template with a total of 45 cycles. Relative expression was calculated by the 2^−ΔΔCt^ method. The *β-actin* gene was used as the internal reference. Primer sequences are shown in Table 1.

**Table 1:** Real-time RT-PCR primers

Primer Name	Upstream	Downstream
integrin alpha7B	gttgtggaaggagtccc	gtcttcccgagggatctt
integrin alpha7X2	ctatccttgcgcagaatgac	gtgaccaacattgatagctc
integrin beta1D	ttgtggagactccagactgtcctact	tcattttccctcatacttcggatt
β-actin	ggg aaa tcg tgc gtg aca tt	gcg gca gtg gcc atc tc

### Co-localization of laminin 2 and integrin β1

The isolated gastrocnemius tissue specimens were fixed with fixative, dehydrated, embedded, sliced, and then stained with a double fluorescence staining kit. All tissue was first observed at a magnification of 100×, and then images were collected at 200× and 400× magnifications, with a total of three fields of view. The fluorescence intensity and the area of all collected images were measured by ImageJ. The average fluorescence intensity (mean gray value) of each image was calculated, and the average fluorescence intensity of two images was averaged to obtain the average fluorescence intensity value of each sample.

### Statistical analysis

Experimental data are expressed as mean ± standard deviation (SD). Statistical analysis was performed using SPSS 25.0 software. When the data of each group conformed to a normal distribution, a one-way analysis of variance was used for comparison between groups, and the Brown–Forsythe test and Bartlett’s test were used to test for homogeneity of variance. When the variances were homogeneous, the LSD test was used; when the variances were unequal, Tamhane’s T2 test was used. *P* < 0.05 was considered statistically significant.

## Results

### Effect of massage on isotonic and tetanic contractility of skeletal muscle in rats undergoing long-term heavy-duty exercise

**Fig. 2:**
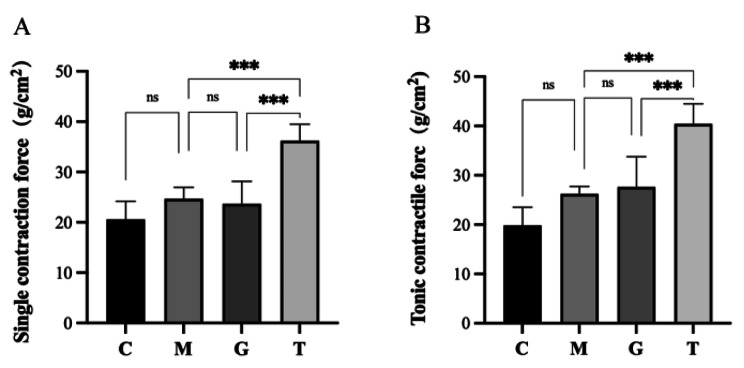
Maximum single contraction force and tetanic force of the medial head of the gastrocnemius muscle in rats. **(A)** Maximum single contraction force. **(B)** Maximum forced contraction force. C, control group; M, long-term heavy-duty exercise group; G, long-term heavy-duty exercise + sham massage group; T, long-term heavy-duty exercise + massage group. ***P < 0.001

Physiological signal acquisition systems were used for ex vivo testing of the contractility of the gastrocnemius muscle. The results show that the single and tetanic contractility of the gastrocnemius muscle after massage intervention was significantly higher than those of other groups (*P* < 0.001). This was not significantly increased in the long-term heavy-duty exercise group compared with the blank group (*P* > 0.05). Therefore, these results show that massage intervention in long-term heavy-duty exercise is effective in promoting increased muscle strength (Fig. 2).

### Effect of massage on the ultrastructure of skeletal muscle in rats undergoing long-term heavy-duty exercise

**Fig. 3:**
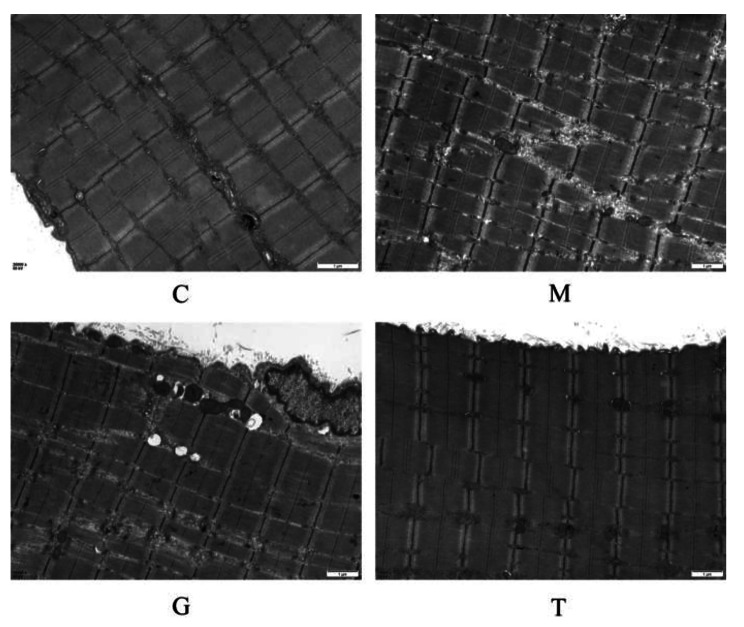
Changes in ultrastructure of the gastrocnemius muscle

Consistent with previous studies, four weeks of continuous eccentric downhill running could lead to skeletal muscle overuse injury, including the accumulation of skeletal muscle cell ultrastructural damage and perimysial fibrosis. In group C, the gastrocnemius muscle myofibrils were neatly arranged, the sarcomere was light, dark bands were clear, mitochondria were regularly arranged on both sides of the Z-line, and the structure was complete. In group M, the Z-line rupture occurred, the mitochondrial structure and the sarcoplasmic reticulum were swollen, and the mitochondrial arrangement was irregular, indicating that the ultrastructure of skeletal muscle was damaged by long-term heavy-duty exercise. Compared with group M, the ultrastructure of the gastrocnemius muscle in group G also showed similar pathological changes. The ultrastructural performance of the gastrocnemius muscle in group T was similar to that in group C, myofibrils and Z-lines were neatly arranged, the sarcoplasmic reticulum was intact, the light and dark bands were clear, and only a few mitochondrial structures were unclear, suggesting that massage intervention can protect bones under long-term heavy-duty exercise. Moreover, massage can protect muscles from damage or promote their repair (Fig. 3).

### Expression of FAK, p-FAK, integrin α7, and integrin β1

**Fig. 4:**
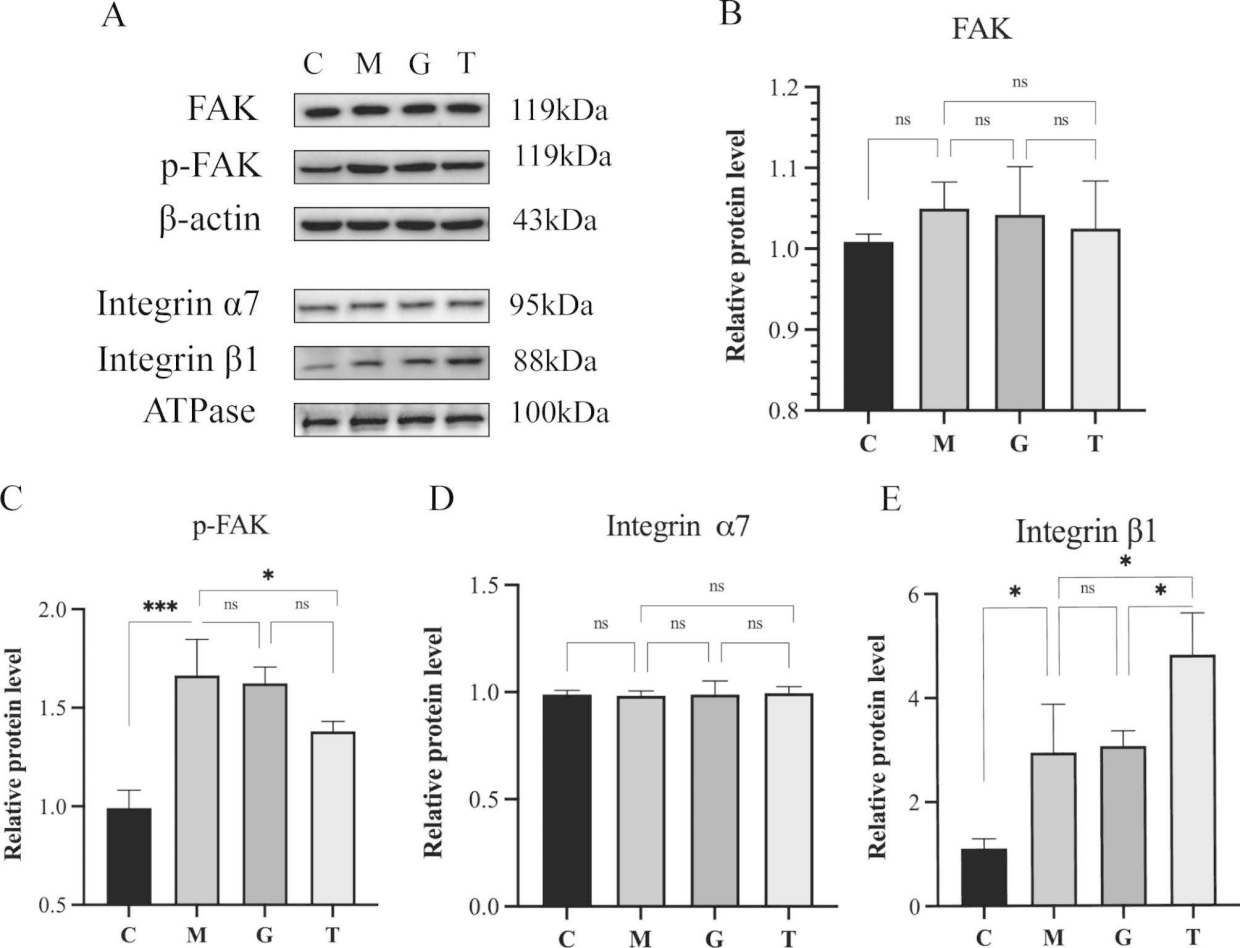
Protein expression in the gastrocnemius muscle of rats. The FAK, p-FAK, β-actin experiments were conducted under the same conditions as each other, and the integrin α7, integrin β1, and ATPase experiments were also conducted under the same conditions as each other. C, control group; M, long-term heavy-duty exercise group; G, long-term heavy-duty exercise + sham massage group; T, long-term heavy-duty exercise + massage group. *P < 0.05, ***P < 0.001

Focal adhesion kinase (FAK) is involved in cell spreading and migration [11] and plays a key role in regulating muscle satellite cell (SC) migration and adhesion, which are essential for wound healing and regeneration of damaged muscle [12]. In vitro studies have also shown that injured tissue can accelerate wound healing by activating the FAK/ERK1/2 signaling cascade through the secretome [13]. It can be seen that the activation of FAK in EIMD is mainly involved in the repair of skeletal muscle, while integrin α7 and integrin β1 are involved in the protection of skeletal muscle. We detected the expression of FAK and integrins by western blot (Fig. 4). The overuse injury model showed increased FAK and p-FAK expression. There was no significant difference in FAK expression, but p-FAK expression showed an extremely significant difference (*P* < 0.001). Massage intervention decreased the expression of FAK protein and significantly decreased the protein expression of p-FAK (*P* < 0.05). Consistent with previous reports [14], the expression level of integrin α7 remained unchanged after repeated supernegative exercise and 24 h after exercise, and massage had no effect on it. However, overuse injury increased the protein expression of integrin β1 (*P* < 0.05), and massage significantly increased the protein expression of integrin β1 in the damaged gastrocnemius sarcolemma (*P* < 0.05). Therefore, we speculate that upon long-term overload exercise, skeletal muscle injury occurred in the model group, and it was repaired by FAK activation, while tuina protected skeletal muscle by increasing the expression of integrin β1.

### mRNA expression of integrins α7B, α7X2, and β1D in gastrocnemius muscle tissue

During skeletal muscle injury repair, subunits of integrin α7β1 are alternatively spliced with developmentally regulated variants, where α7A and α7B, β1A and β1D are intracellular domain variants, and α7X1 and α7X2 are extracellular domain variants [15]. Among them, α7A and α7X1 have specific roles during the dynamic phase of adhesion, while α7B, α7X2, and β1D are predominant during stable adhesion. At the same time, α7X2 is also highly expressed during late repair [15], and usually β1D interacts with the actin cytoskeleton more strongly than β1A [6], while β1D upregulation seems to be associated with new tendon junctions. The formation of tau occurs in parallel and serves to transmit tension from the cytoskeleton to the ECM, which can serve as a marker of firm adhesion to the ECM [15]. To clarify the effect of tuina on cell adhesion function during long-term overload exercise, we determined the mRNA levels of the major integrin subunits that affect cell stable adhesion function to judge the state of muscle defense against injury.

**Fig. 5:**
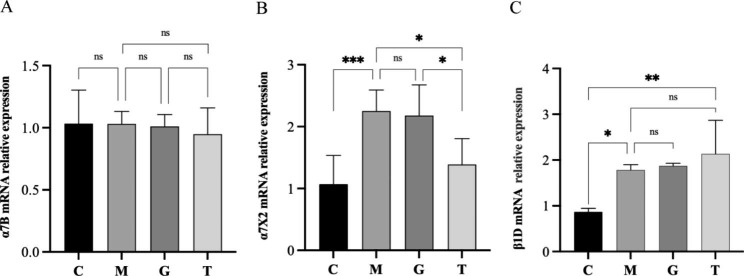
Relative mRNA expression in gastrocnemius muscle tissue of rats. C, control group; M, long-term heavy-duty exercise group; G, long-term heavy-duty exercise + sham massage group; T, long-term heavy-duty exercise + massage group. *P < 0.05, **P < 0.01, ***P < 0.001

There was no significant difference in the mRNA expression of integrin α7B in gastrocnemius muscle tissue of rats between the C, M, G, and T groups (Fig. 5). Compared with group C, the mRNA expression of integrin α7X2 in gastrocnemius muscle tissue of group M was significantly higher (*P* < 0.001); compared with group M, there was no significant difference in the mRNA expression of integrin α7X2 in group G; the mRNA expression of integrin α7X2 in group T was lower (*P* < 0.05). Compared with group C, the mRNA expression of integrin β1D in gastrocnemius muscle tissue of rats in group M was significantly higher (*P* < 0.05); compared with group M, there was no significant difference in the mRNA expression of integrin β1D in group G; the mRNA expression of integrin β1D in group T was significantly higher (*P* < 0.01).

### Co-localization of laminin 2 + integrin β1 in gastrocnemius muscle tissue of rats

Integrin α7β1 interacts with laminin 2, regulates mechanotransduction, and prevents skeletal muscle injury [5]. To explore the interaction between laminin 2 and integrin β1 in the cell membrane, we conducted an immunofluorescence analysis (Table 2). Compared with group C, the contents of laminin 2, integrin β1, and the co-expressed proteins of laminin2 + integrin β1 in the gastrocnemius muscle tissue of the rats in group M were significantly increased (*P* < 0.05 and *P* < 0.01). Compared with group C, the contents of laminin 2 protein and the co-expressed proteins of laminin 2 + integrin β1 in group G were significantly increased (*P* < 0.05), while the expression of integrin β1 tended to increase, though there was no statistical difference (*P* > 0.05). Compared with group C, the contents of laminin 2, integrin β1 protein, and co-expressed proteins of laminin 2 + integrin β1 in group T were significantly increased (*P* < 0.01). There was no significant difference between group M and group G (*P* > 0.05) in terms of the contents of laminin 2, integrin β1, and the co-expressed proteins of laminin2 + integrin β1 in the gastrocnemius muscle tissue of rats. Compared with group M, the contents of laminin 2, integrin β1 protein, and the co-expressed proteins of laminin 2 + integrin β1 in group T were significantly increased (*P* < 0.01).

**Table 2:** Mean fluorescence intensity of co-localization of laminin 2 and integrin β1 (± SD).

Group	Number	Laminin 2 Mean (± SD)	Integrin β1 Mean (± SD)	Laminin 2 + Integrin β1 Mean (± SD)
C	3	22.8622 ± 2.4773	21.4190 ± 2.8024	19.9190 ± 1.7271
M	3	29.1192 ± 3.1710*	29.2802 ± 3.5792*	30.0422 ± 4.1885**
G	3	29.3700 ± 4.4118*	28.1835 ± 5.0830	28.1755 ± 5.2466*
T	3	40.0330 ± 2.6758**^##^	40.6778 ± 3.3423**^##^	41.3557 ± 1.0170**^##^

*P < 0.05, **P < 0.01 compared with group C; ^##^*P* < 0.01 compared with group M

**Fig. 6:**
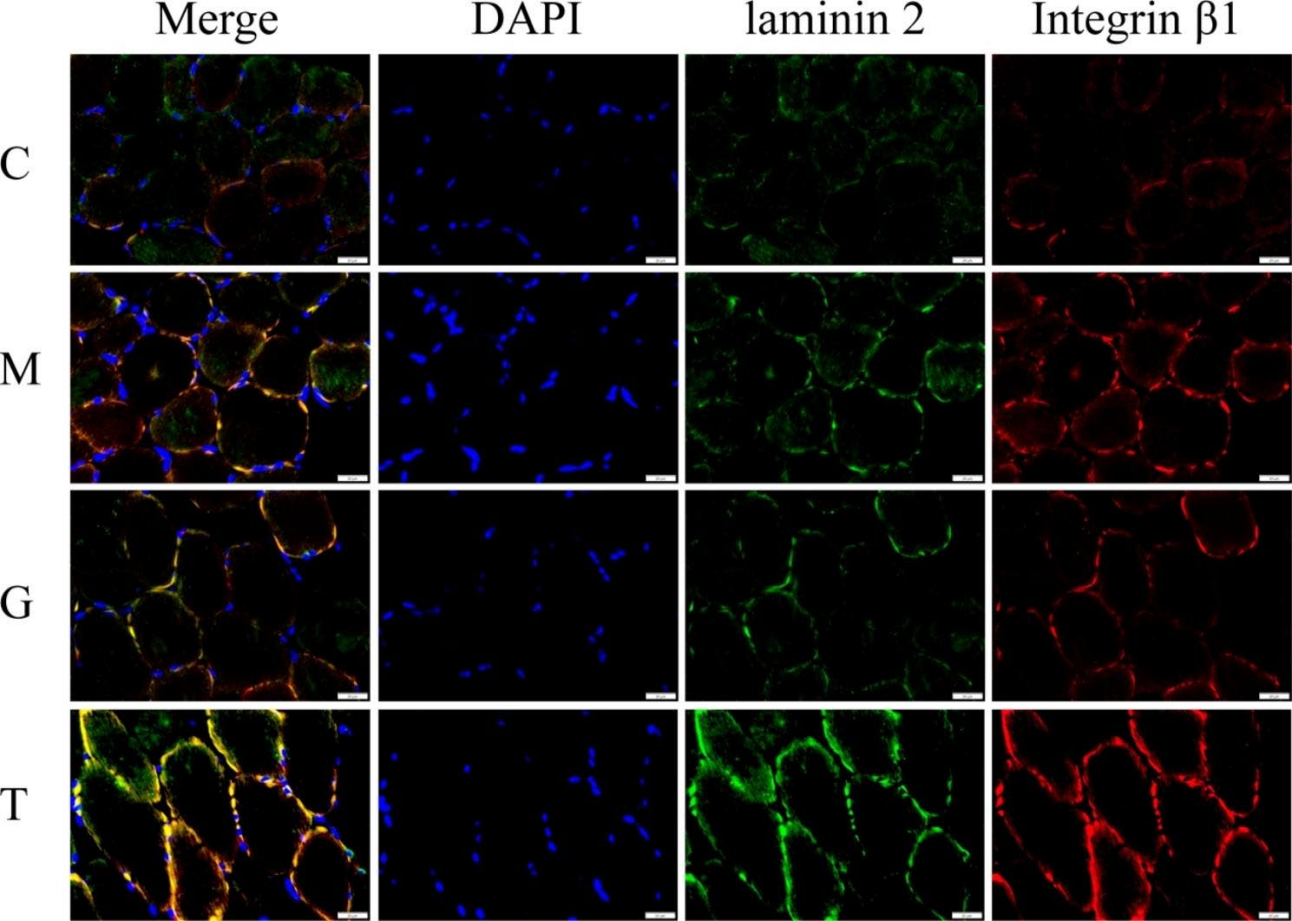
Immunofluorescence staining

The immunofluorescence staining results are shown in Fig. 6, where DAPI-stained nuclei are shown in blue, laminin 2 is shown in green, and integrin β1 is shown in red.

## Discussion

As a mechanical therapy, massage is widely used in sports training. Massage improves muscle properties and promotes recovery after repetitive muscle contractions [16, 17]. Currently, there are challenges in research on massage. The main challenge is that there are differences in the frequency and intensity of different operators and different massage techniques, and this difference has an impact on the research results. Therefore, we used FSR film. The pressure sensor monitors the frequency and strength of massage in real time, ensuring the constant recording of massage parameters.

The phenomenon of skeletal muscle adaptation to long-term overload exercise and protecting muscle from injury is called the repeated bout effect (RBE). The main mechanisms include neural adaptation, changes in muscle mechanical properties, structural remodeling of the ECM, and modified inflammatory responses [18]. Among them, the structural remodeling of the ECM, which is a complex network composed of collagen, proteoglycan, and laminin that wraps skeletal muscle fibers, has a more durable protective effect on muscle tissue. The ECM is involved in cell adhesion and intercellular signaling, plays an important role in the stability of the internal environment [19], and can also buffer muscle fibers from mechanical strain by increasing passive tension, thereby protecting muscles from injury [18]. In the present study, we found that massage can increase the benefits of long-term overload exercise on muscle strength, and at the same time massage amplifies the RBE, avoids damage to skeletal muscle caused by long-term overload, and ensures sarcolemma and muscle fiber arrangement and stability.

Integrins are transmembrane heterodimeric cell adhesion molecules composed of two non-covalently bound alpha and beta transmembrane subunits. Of particular relevance in adult skeletal muscle are integrins α7 and β1, with integrin α7 predominantly expressed on muscle fibers at the tendon junction, with weak and irregular expression on muscle belly fibers. Integrin β1 is expressed in endothelial cells, muscle fibers, and peripheral nerves, and its expression is increased after crush injury, whereas integrin α7 expression is not increased in injured muscle fibers [20]. The actin cytoskeleton is linked to the ECM by integrins to perform cell adhesion functions. In addition to fixing cells, integrin adhesion can also work together in the form of mechanotransduction and signaling pathways, providing sensory input to cells and protecting cells. Skeletal muscle provides the foundation [5]. Increasing the number of β1D chains in muscular dystrophy mdx mice [21] also increases the expression of α7, and in vitro experiments have also demonstrated that increasing the number of β1D chains enhances the transcription of integrin α7 and laminin α2 genes and the amount of protein [8]. All this evidence suggests that integrin β1D plays an important role in the protective mechanism of skeletal muscle. We found that massage intervention after long-term overload exercise can increase the expression of the membrane protein integrin β1 and the basement membrane laminin 2, thereby protecting skeletal muscle from injury.

## Conclusions

In previous studies, the application of Chinese massage was mostly used for the treatment of existing lesions and injuries. This experiment demonstrates, for the first time, that compression techniques in massage can prevent skeletal muscle injury in long-term heavy-duty sports and enhance the benefits of sports training on muscle function. In addition, massage can protect skeletal muscle from injury by upregulating integrin β1 and basement membrane layer adhesion protein 2 to prevent overuse injury, thus enhancing cell adhesion function. This study provides a methodological and theoretical basis for activities such as competitive sports training and heavy physical work. It also expands the scope of application of TCM massage and complements the theoretical basis of TCM massage in complementary and alternative medicine. In future studies, we can investigate whether the findings that massage protects skeletal muscle from injury in human trials with different massage parameters (e.g. frequency, intensity and duration of intervention) are equally valid, as well as further explore the deeper role of the relationship between integrins and ECM in the mechanism of skeletal muscle protection and the possibility that the protective mechanism of massage on skeletal muscle is not only through integrins and laminin. Perhaps there are more pathways of action waiting to be discovered.

## Electronic supplementary material

Below is the link to the electronic supplementary material.

Supplementary Material 1

## Data Availability

The datasets used and/or analyzed during the current study are available from the corresponding author on reasonable request.
