# Supplementary material for: Massage protects skeletal muscle from injury during long-term heavy-duty exercise via integrin β1 and laminin 2 channels of basement membrane
Source: BMC Complement Med Ther. 2023 Jul 26;23:266. doi: 10.1186/s12906-023-04094-6 (PMC10369692; doi:10.1186/s12906-023-04094-6)
Supplement: Supplementary file 1 — Supplementary Material 1 [file 12906_2023_4094_MOESM1_ESM.docx]

Supplementary Material

1. Western Blot Original Picture

| **FAK**  The groups are CMGTCMGTCMGT | **p-FAK**  The groups are CMGTCMGTCMGT |
| --- | --- |
| 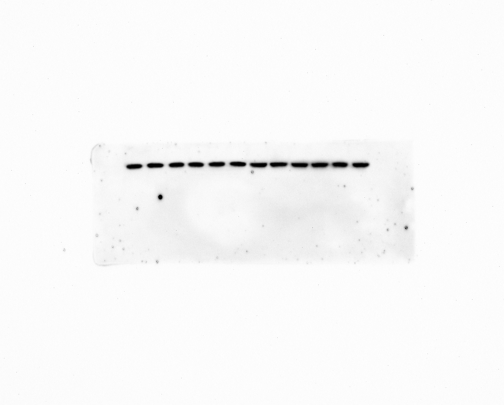  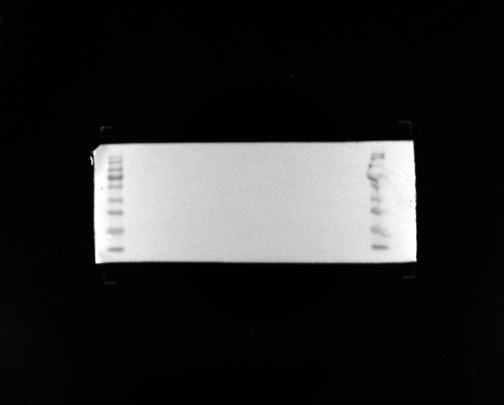 | 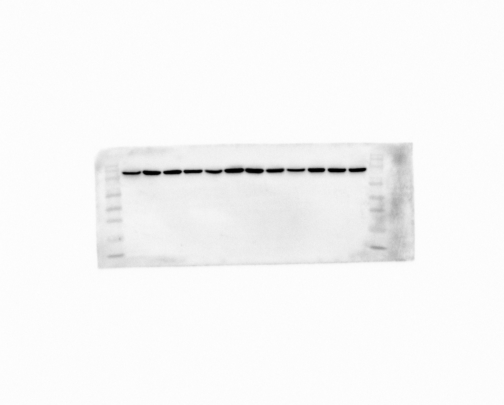  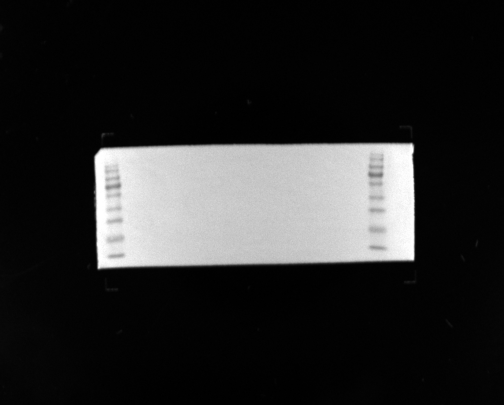 |
| **β-actin**  The groups are CMGTCMGTCMGT | **Integrin α7**  The groups are CMGTCMGTCMGT |
| 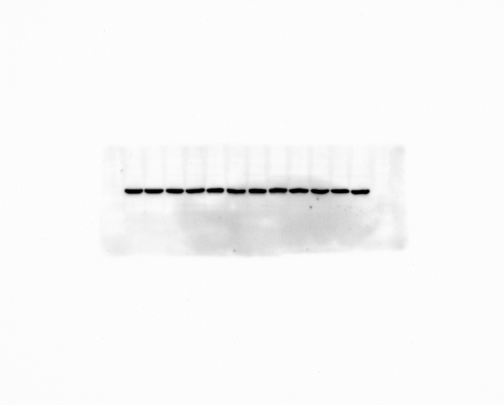  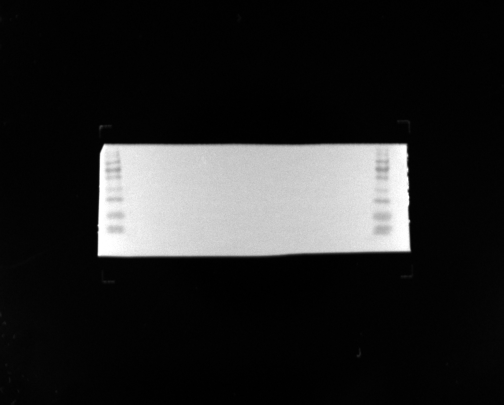 | 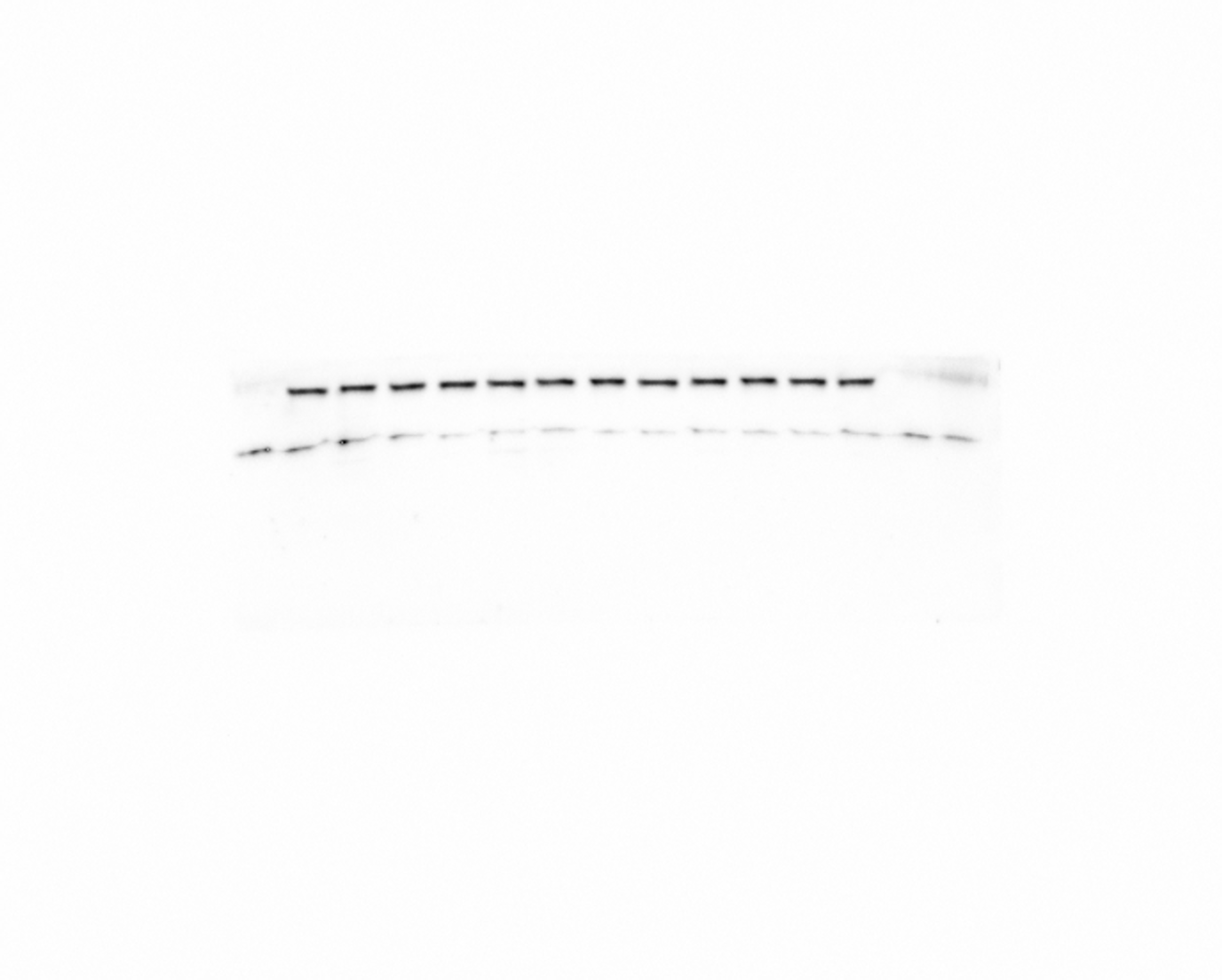  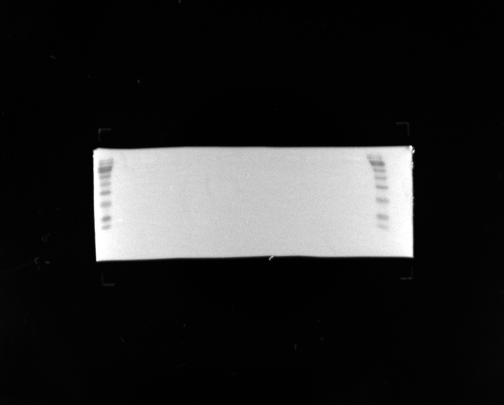 |
| **Integrin β1**  The groups are CMGTCMGTCMGT | **Sodium Potassium ATPase**  The groups are CMGTCMGTCMGT |
| 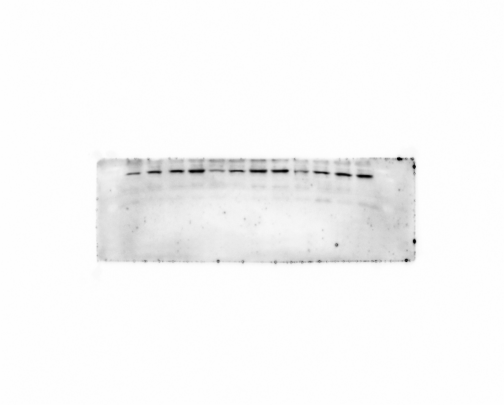  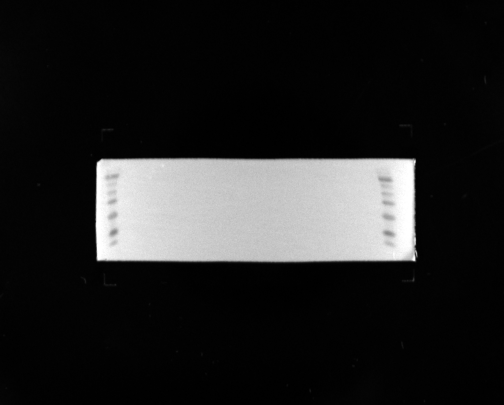 | 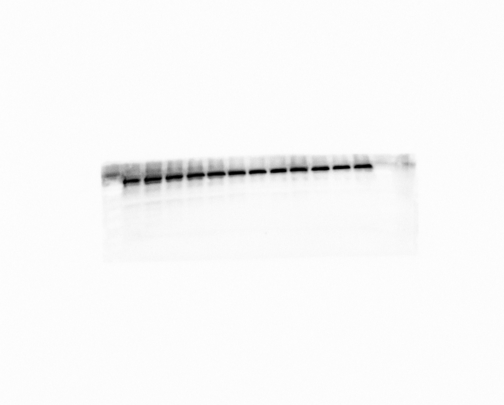  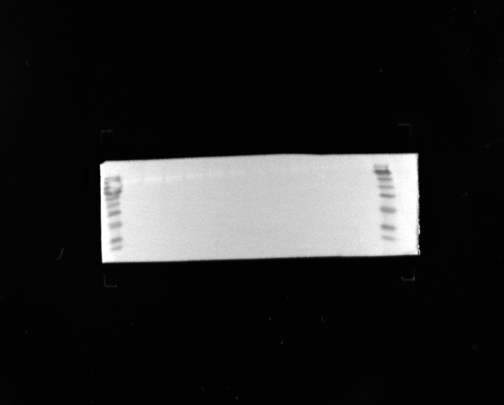 |

1. Western Blot Original IOD value

| **Groups** | **C** | **M** | **G** | **T** | **Groups** | **C** | **M** | **G** | **T** |
| --- | --- | --- | --- | --- | --- | --- | --- | --- | --- |
| FAK | 766.75 | 817.52 | 749.67 | 729.27 | p-FAK | 551.47 | 872.98 | 837.76 | 728.83 |
|  | 780.32 | 803.82 | 830.13 | 823.47 |  | 593.81 | 1069.30 | 997.20 | 790.67 |
|  | 810.61 | 855.25 | 841.48 | 807.28 |  | 520.56 | 883.44 | 883.57 | 766.76 |
| **Groups** | **C** | **M** | **G** | **T** | **Groups** | **C** | **M** | **G** | **T** |
| β-actin | 1014.80 | 1008.80 | 997.43 | 1006.30 | Integrin α7 | 172.40 | 178.33 | 171.43 | 180.90 |
|  | 1013.70 | 1051.20 | 1073.20 | 1016.80 |  | 170.99 | 172.84 | 178.23 | 176.13 |
|  | 1065.10 | 1063.30 | 1004.70 | 1022.90 |  | 176.86 | 171.91 | 164.99 | 181.46 |
| **Groups** | **C** | **M** | **G** | **T** | **Groups** | **C** | **M** | **G** | **T** |
| Integrin β1 | 77.78 | 187.99 | 219.64 | 443.76 | ATPase | 767.85 | 788.86 | 774.55 | 781.94 |
|  | 83.56 | 180.17 | 276.61 | 400.63 |  | 786.39 | 784.30 | 753.10 | 799.99 |
|  | 105.30 | 199.84 | 250.63 | 406.26 |  | 787.10 | 796.65 | 791.76 | 828.09 |
